# Diet Quality, Food Processing, and Nutrient Adequacy in Vegan, Vegetarian, and Omnivorous Dietary Patterns: A Critical Narrative Review

**DOI:** 10.3390/nu18142291

**Published:** 2026-07-13

**Authors:** Vicente Javier Clemente-Suárez, Edgar Simon Sancho-Haro, Rodrigo Yáñez-Sepúlveda, José Francisco Tornero-Aguilera, Alexandra Martín-Rodríguez

**Affiliations:** 1 Department of Sport Sciences, Faculty of Sport and Health Sciences, Fit Generation Research Institute, AD400 Andorra la Vella, Andorra; jtornero@fitgeneration.es; 2 Grupo de Investigación en Cultura, Educación y Sociedad, Universidad de la Costa, Barranquilla 080002, Colombia; 3 Department of Nutrition and Dietetics, Faculty of Sport and Health Sciences, Fit Generation Research Institute, AD400 Andorra la Vella, Andorra; edgar.sancho@fitgeneration.es; 4 Faculty of Education and Social Sciences, Universidad Andrés Bello, Viña del Mar 2520000, Chile; rodrigo.yanez@unab.cl; 5 School of Medicine, Universidad Espíritu Santo, Samborondón 091650, Ecuador; 6 Faculty of Education Sciences, UNIE University, 28015 Madrid, Spain; sandra.martin.rodriguez8@gmail.com

**Keywords:** cardiometabolic health, comparator quality, dietary patterns, food processing, Mediterranean vegan, nutrient adequacy, omnivorous diet, plant-based diet, vegan diet, vegetarian diet

## Abstract

Vegan and vegetarian dietary patterns have moved from minority dietary choices to central elements of contemporary nutritional guidance, supported by observational cohorts, short-term randomised trials, and umbrella-level evidence (i.e., syntheses of multiple systematic reviews) reporting favourable associations with body weight, blood lipids, blood pressure, glycaemic control, and inflammatory markers. Recent direct comparisons against healthy omnivorous comparators—including the OMNIVEG controlled crossover transitioning from a traditional to a vegan Mediterranean diet and the Landry identical-twin trial—indicate that well-formulated plant-based patterns can produce additional short-term improvements in selected cardiometabolic markers even when the comparator is not the modern Western diet. Interpretation of the broader evidence base is nonetheless constrained by comparator quality, residual confounding, exposure misclassification, reliance on intermediate biomarkers, and limited follow-up. This critical narrative review integrates clinical, methodological, nutritional, and physiological evidence to compare vegan, vegetarian, and omnivorous dietary patterns, and argues that the dominant explanatory axis of long-term outcomes is dietary pattern quality—minimally processed plant-rich composition, nutrient adequacy, low ultra-processed food intake, and adherence—rather than the binary inclusion or exclusion of animal-source foods. Well-planned vegan and vegetarian patterns are valid dietary expressions that require systematic attention to vitamin B12, iodine, iron, zinc, calcium, vitamin D, EPA/DHA, and protein quality. Public health messaging should prioritise food quality, processing level, nutrient adequacy, adherence, and outcome hierarchy over binary dietary identities.

## 1. Introduction

Plant-based dietary patterns have moved from being minority dietary choices to central components of contemporary nutritional guidelines, clinical recommendations, and public health discourse. Vegetarian diets usually exclude meat and fish but may include eggs and dairy products, whereas vegan diets exclude all animal-derived foods. These patterns are promoted on the basis of cardiometabolic health, body weight control, environmental sustainability, and ethical considerations, and the strongest institutional position is that appropriately planned vegetarian dietary patterns, including vegan diets, can be nutritionally adequate and may provide health benefits in the prevention and management of selected chronic diseases [1,2]. More recent position statements have adopted a more nuanced tone, emphasising the need for careful planning, reliable vitamin B12 supplementation, and particular attention in physiologically vulnerable groups [3]. Nutritional adequacy under conditions of careful supplementation and dietary planning is therefore the established baseline; the unresolved scientific question is comparative rather than categorical.

The evidence supporting vegetarian and vegan diets derives mainly from prospective cohort studies, systematic reviews, meta-analyses, umbrella reviews (syntheses of systematic reviews), and a growing number of randomised clinical trials. Recent umbrella-level evidence indicates that vegetarian dietary patterns, including vegan diets, are associated with lower cardiovascular disease incidence and mortality and with reductions in blood pressure, LDL cholesterol, body mass index, and inflammatory markers compared with non-vegetarian dietary patterns [4]. Large prospective cohorts—particularly the Adventist Health Study-2 and EPIC-Oxford—have provided the epidemiological scaffolding for this favourable interpretation [5,6]. Yet the picture is more nuanced than it appears at first sight: EPIC-Oxford, while reporting lower ischaemic heart disease in vegetarians and fish eaters, simultaneously documented higher rates of total and haemorrhagic stroke and a measurably increased fracture risk in vegans, suggesting that the cardiometabolic and skeletal signatures of vegan dietary patterns are not uniformly favourable across all clinical domains.

A pivotal interpretive issue across the broader literature is the quality of the comparator diet. Much of the apparent advantage of vegan or vegetarian diets in older cohorts reflects comparison against typical Western omnivorous diets characterised by higher intake of refined grains, processed meats, added sugars, excess energy, alcohol, and ultra-processed foods. Any dietary pattern that eliminates these features is likely to improve cardiometabolic markers irrespective of animal-food inclusion. The conceptual distinction between plant-based diet quality and animal-food exclusion is illustrated by prospective evidence showing that healthful plant-based diet indices (hPDIs) are associated with lower coronary heart disease risk, whereas unhealthful plant-based diet indices (uPDIs) rich in refined grains, sweets, and sugar-sweetened beverages are associated with higher risk [7]. More recent analyses in the UK Biobank confirm that healthful plant-based diets (high hPDI) are associated with lower mortality and a lower risk of cancer and cardiovascular disease, while ultra-processed plant-based diets show opposite associations [8,9]. In the French NutriNet-Santé cohort, the balance between animal- and plant-based foods, the nutritional quality of the diet, and the food processing level were each independently associated with cardiovascular risk [10]. Together, these data invite a reformulation of the central question: not whether vegan diets are better than omnivorous ones, but which combination of pattern quality, processing level, and nutrient adequacy best supports long-term health.

A second pillar of the comparison is nutritional adequacy. Vegan diets typically provide higher intakes of fibre, folate, vitamin C, vitamin E, magnesium, and a broad portfolio of phytochemicals, and lower intakes or status of vitamin B12, vitamin D, iodine, calcium, EPA, DHA, iron, zinc, and sometimes total protein compared with meat-containing diets [11]. Vitamin B12 is the clearest example, because it is not reliably supplied by unfortified plant foods and requires supplementation or fortified foods in strict vegan diets [12]. Fortification and supplementation are normal tools of contemporary public health nutrition; the appropriate framing is therefore not biological inferiority but the systematic attention to nutrients of concern that planned vegan diets require [3]. Bioavailability further qualifies the picture: plant foods contain food matrix components that modulate mineral absorption [13], and contemporary trials suggest physiological adaptation in long-term vegans that partially offsets the lower theoretical bioavailability of non-haem iron [14]. Protein quality assessment has been refined through the digestible indispensable amino acid score (DIAAS) [15], and recent randomised work shows that well-balanced vegan diets do not compromise daily mixed muscle protein synthesis in active older adults [16] and that plant-based dietary patterns do not impair muscular strength compared with omnivorous patterns in randomised trials [17].

Human dietary evolution is best characterised by ecological flexibility rather than by any single ancestral diet [18,19]. Animal-source foods have historically contributed protein, vitamin B12, retinol, haem iron, zinc, long-chain omega-3 fatty acids, and bioactive compounds such as taurine, creatine, and carnosine [20]; cooked starch-rich plant foods, fruits, seeds, nuts, honey, and fermented preparations have been equally central. Evolutionary biology supports humans as flexible omnivores, but it does not demonstrate the superiority of any contemporary dietary pattern in modern societies where fortification, supplementation, year-round food availability, and rigorous nutritional knowledge provide tools that did not exist in ancestral contexts. The Mediterranean pattern further illustrates that a plant-rich, minimally processed omnivorous diet can reduce hard cardiovascular endpoints in randomised trials (PREDIMED, CORDIOPREV) [21,22]; CARDIVEG and Barnard 2022 show that vegan and Mediterranean patterns can produce comparable or differential cardiometabolic effects across selected markers [23,24]. Crucially, the OMNIVEG controlled crossover trial demonstrated that transitioning from a traditional Mediterranean diet to a vegan Mediterranean diet in healthy active men reduced total cholesterol and LDL-C without compromising fat oxidation, the exercise intensity at which fat oxidation is maximal (Fatmax), energy expenditure, heart rate, or rating of perceived exertion during progressive exercise [25,26], and the Landry identical-twin trial reported greater reductions in LDL cholesterol, fasting insulin, and body weight in participants assigned to a healthy vegan diet compared with those assigned to a healthy omnivorous diet [27]. These designs compare two high-quality dietary patterns rather than a “good” diet to a “poor” one and therefore inform the comparative question more directly than earlier observational work.

Accordingly, the present review integrates the clinical, methodological, nutritional, physiological, and contextual evidence comparing vegan, vegetarian, and omnivorous dietary patterns. The central question is not whether vegan and vegetarian diets can be healthy when carefully planned—the evidence indicates that they can—but whether dietary pattern quality, processing level, nutrient adequacy, and adherence explain more variance in long-term outcomes than the categorical presence or absence of animal-source foods. The review proceeds by appraising the principal evidence base from cohort and umbrella-level studies, examining randomised comparisons against healthy omnivorous comparators, dissecting the methodological constraints that condition interpretation, integrating food matrix biology with nutrients requiring systematic attention, and proposing a pattern quality framework that aligns clinical practice and public health messaging with the most defensible reading of contemporary evidence.

## 2. Materials and Methods

This article was designed as a critical narrative review of the clinical, methodological, nutritional, and contextual evidence comparing vegan, vegetarian, and omnivorous dietary patterns. The review is explicitly narrative and non-systematic; it does not claim PRISMA 2020 compliance, no de novo meta-analysis was performed, and no PRISMA flow diagram, duplicate screening, or reproducible database-specific search strategy was implemented. Where comparative evidence was synthesised without quantitative pooling, the SWiM (Synthesis Without Meta-analysis) framework [28] was used as a reporting guide, and the SANRA scale for the quality of narrative reviews [29] was applied internally to support reporting transparency rather than as an external quality warrant. A structured literature search was performed in PubMed/MEDLINE, Scopus, Web of Science, and the Cochrane Library up to April 2026, complemented by reference list screening of relevant systematic reviews, meta-analyses, cohort studies, randomised trials, and position statements. Search terms covered vegan, vegetarian, plant-based, whole-food plant-based and vegan Mediterranean diets; cardiovascular disease, mortality, type 2 diabetes, cancer, fracture risk, bone mineral density, muscle protein synthesis and muscular strength; vitamin B12, iron, zinc, iodine, calcium, vitamin D, EPA, DHA, protein quality and DIAAS; phytate, food matrix, food processing, NOVA, hPDI and uPDI; and Mediterranean diet, OMNIVEG, and dietary pattern quality. Studies published from January 2000 onward were prioritised, with greater interpretative weight given to randomised trials, large prospective cohorts, and systematic reviews published since 2018.

Studies were selected when they contributed directly to the comparative evaluation of vegan, vegetarian, or omnivorous diets in relation to human health, nutritional adequacy, or biological plausibility, and excluded when they were not conducted in humans, when the dietary exposure was insufficiently defined, when the intervention combined multiple lifestyle components in a way that prevented isolation of the dietary component, or when they focused exclusively on environmental or ethical arguments without direct relevance to human physiology, nutrition, or health outcomes. Comparator validity was treated as a primary interpretive criterion: studies that compared vegan or vegetarian diets with the modern Western diet were considered informative for assessing the replacement of low-quality dietary patterns, but not for testing whether animal-food exclusion is comparatively advantageous against equivalently well-formulated omnivorous patterns—an assertion that requires direct comparisons against high-quality, minimally processed omnivorous comparators (CARDIVEG, OMNIVEG). Because this was not a de novo systematic review, no formal GRADE assessment was performed; a narrative per-outcome certainty schema was applied based on consistency, study design, comparator quality, risk of bias, duration, and the distinction between hard endpoints and intermediate biomarkers. The evidence was synthesised narratively rather than pooled quantitatively because of heterogeneity in dietary definitions, populations, comparators, outcomes, follow-up duration, and methodological quality. As a critical narrative rather than systematic review, this work does not apply the PRISMA reporting framework, which is designed for systematic reviews; instead, transparency and rigour were guided by the SANRA quality criteria and by SWiM (Synthesis Without Meta-analysis) principles, adopted because the heterogeneity of study designs, comparators, and outcomes precludes a formal quantitative meta-analysis.

## 3. The Observational and Umbrella-Level Evidence Base

A fair appraisal of vegan and vegetarian dietary patterns must begin by acknowledging the favourable signals that have accumulated across observational, interventional, and umbrella-level evidence. The 2024 umbrella review by Landry et al. [4] synthesised systematic reviews across more than two decades of prospective and interventional data and reported lower cardiovascular incidence and mortality, lower LDL cholesterol, and reductions in blood pressure and body mass index in vegetarian dietary patterns. EPIC-Oxford reported lower rates of ischaemic heart disease in fish eaters and vegetarians than in meat eaters over 18 years of follow-up [6]; the Adventist Health Study-2 documented lower all-cause and selected cause-specific mortality in vegetarian dietary patterns [5]. These cohorts, alongside the French NutriNet-Santé and the UK Biobank, provide the epidemiological scaffolding for the favourable interpretation of plant-rich diets, but they also illustrate the constraints of observational nutrition science: dietary patterns are self-selected, healthy-volunteer bias is pervasive, food frequency questionnaires limit exposure resolution, and the boundary between “plant-based” and “ultra-processed plant-based” is poorly captured by traditional dietary assessment instruments. To give the reader a quantitative sense of magnitude, meta-analyses estimate that vegetarian and vegan patterns lower LDL cholesterol by roughly 0.3–0.5 mmol/L (approximately 12–20 mg/dL) relative to omnivorous diets, and prospective cohorts report all-cause mortality hazard ratios in vegetarians on the order of 0.88–0.91; these estimates nonetheless carry wide confidence intervals and substantial between-study heterogeneity, and should be read as indicative rather than definitive effect sizes.

Mechanistically, the LDL cholesterol signal is plausibly mediated by higher intakes of soluble fibre (particularly β-glucan from oats and barley), plant sterols, monounsaturated fatty acids from nuts and olive oil, and reductions in saturated fatty acid intake; soluble fibre attenuates intestinal cholesterol and bile acid reabsorption and increases hepatic LDL receptor expression, plant sterols compete with cholesterol for incorporation into mixed micelles, and reductions in saturated fatty acids decrease hepatic LDL receptor down-regulation [30]. The blood pressure signal has been attributed to a higher potassium-to-sodium ratio, higher intake of nitrate-rich vegetables, and weight loss—mechanisms shared with the Mediterranean and DASH dietary patterns rather than unique to animal-food exclusion [31]. Vegan and vegetarian diets also tend to reduce body weight in short-term trials and are associated with lower BMI in cross-sectional and prospective data [4,27], an effect plausibly mediated by lower energy density, higher fibre, and spontaneous reductions in energy intake when ultra-processed foods are simultaneously limited. The 8-week identical-twin randomised trial by Landry et al. [27] reported a mean body-weight reduction of approximately 2 kg in the vegan arm relative to the omnivorous arm under dietitian-supported guidance for both groups.

For type 2 diabetes and glycaemic control, plant-rich patterns are associated with lower incidence in prospective cohorts and with improved glycaemic markers in short-term controlled trials [4,11], with effect magnitudes that depend on baseline glycaemic status, weight loss, and dietary adherence. The distinction between whole-food plant-based diets and diets dominated by refined carbohydrates and ultra-processed plant products is particularly consequential for glycaemic outcomes [7,8,9]: the former consistently improve postprandial glucose, fasting insulin, and homeostatic model assessment of insulin resistance (HOMA-IR) in short-term trials [24], whereas the latter tend to show neutral or unfavourable effects despite a nominally plant-based label. The biological plausibility of the glycaemic signal rests on three convergent mechanisms: lower hepatic and intramyocellular lipid accumulation with reductions in saturated-fat intake; improved insulin sensitivity associated with weight loss and increased fibre intake; and beneficial shifts in gut microbial composition and short-chain fatty acid production [32,33]. Direct comparative evidence against high-quality omnivorous comparators on hard endpoints remains limited, and observational associations cannot be uncritically translated into causal inference.

Associations between vegetarian or vegan diets and cancer risk are heterogeneous across cancer types, dietary subgroups, and analytic approaches. Recent pooled analyses across multiple prospective cohorts and a 2026 systematic review and meta-analysis suggest a modest reduction in overall cancer incidence among vegetarian and vegan dietary patterns, with site-specific signals that vary in magnitude and consistency [34,35]. These findings are compatible with several mechanisms—lower processed meat intake, higher fibre intake, lower adiposity, higher intake of phytochemical-rich foods, and differences in alcohol consumption or smoking—yet body weight, screening behaviour, smoking, alcohol intake, socioeconomic position, and broader health-conscious behaviours remain major sources of potential confounding. Current evidence therefore supports a cautious interpretation: vegetarian and vegan dietary patterns may be associated with a lower risk of some cancers, but whether this reflects strict animal-food exclusion, avoidance of processed meat, higher plant food quality, lower BMI, or a combination of these factors cannot be resolved from current epidemiology. Higher fibre, prebiotic carbohydrate, and polyphenol intake associated with plant-rich diets is consistently linked to changes in gut microbiota composition and to increased production of short-chain fatty acids [32]; recent randomised data indicate that high-fibre plant-based interventions can selectively expand butyrate-producing taxa and modestly reduce circulating C-reactive protein and interleukin-6 in metabolically vulnerable populations [32,33], although effect sizes are modest and reversibility upon dietary discontinuation is rarely documented.

Table 1 summarises the principal prospective cohort studies that anchor the observational evidence base for vegan, vegetarian, and plant-based dietary patterns. Three features are noteworthy across these cohorts. First, the most consistent signals across Adventist Health Study-2, EPIC-Oxford, NutriNet-Santé, and the UK Biobank are favourable cardiovascular and metabolic associations for plant-rich patterns, with effect sizes that depend strongly on adherence and baseline diet quality. Second, EPIC-Oxford illustrates the asymmetric clinical signature of strict vegan diets: lower ischaemic heart disease but higher haemorrhagic stroke and increased fracture risk, reminding the reader that “plant-based” is not uniformly favourable across all clinical domains. Third, the contrast between hPDI/uPDI and NOVA food processing analyses (where NOVA is a four-level classification grouping foods from unprocessed to ultra-processed) in the UK Biobank reframes the field: ultra-processed plant-based exposures behave like ultra-processed omnivorous exposures, and dietary pattern quality dominates the variance over the binary inclusion or exclusion of animal foods.

## 4. Randomised Comparisons Against Healthy Omnivorous Comparators

A specific limitation of much of the older vegan/vegetarian diet literature is the use of typical Western omnivorous comparators, which makes apparent differences difficult to attribute to animal-food exclusion as such. A growing body of recent randomised and controlled work addresses this limitation by comparing well-formulated plant-based patterns against equally well-formulated omnivorous patterns, providing the most direct test of comparative superiority that the field currently has. The OMNIVEG controlled crossover trial in 14 healthy physically active men [25] compared a traditional Mediterranean diet (3 weeks) with a vegan Mediterranean diet (4 weeks) separated by a 1-week washout, matched for the principal qualitative features of the Mediterranean pattern; transition from the traditional to the vegan Mediterranean diet reduced total cholesterol and LDL cholesterol. Because the comparator was a high-quality Mediterranean pattern rather than a low-quality Western diet, the trial provides direct evidence that a well-formulated vegan Mediterranean pattern can improve selected cardiometabolic markers relative to a traditional Mediterranean phase under controlled conditions. A companion OMNIVEG analysis on exercise metabolism reported no deterioration in maximal fat oxidation, Fatmax, energy expenditure, heart rate, or rating of perceived exertion during progressive exercise after the same dietary transition [26]. Together, these findings argue against any general claim that the removal of animal foods compromises performance physiology when the dietary pattern is well planned, although the modest sample, the male-only and physically active population, and the short duration constrain external validity and preclude inference about long-term effects.

The Landry et al. randomised trial in 22 pairs of identical twins [27] compared a healthy vegan diet with a healthy omnivorous diet over 8 weeks, with both groups receiving dietitian-supported dietary guidance. The vegan arm showed greater reductions in LDL cholesterol, fasting insulin, and body weight than the omnivorous arm. The within-twin design controls for shared genetic and environmental confounders—a methodological strength that places this trial near the top of the observational-to-experimental continuum—although the short duration, intermediate-biomarker endpoints, and reduced supervision of food intake during the second four weeks limit long-term inference. CARDIVEG, by contrast, compared a lacto-ovo-vegetarian hypocaloric diet with a Mediterranean hypocaloric diet [23] and reported comparable weight loss and reductions in fat mass with differential lipid profiles: the vegetarian arm produced greater LDL cholesterol reductions while the Mediterranean arm produced greater triglyceride reductions. The Barnard 2022 randomised crossover trial [24] compared a low-fat vegan diet with a Mediterranean diet, with the vegan arm producing greater reductions in body weight, lipid markers, and insulin resistance and the Mediterranean arm tending to favour blood pressure. As with all single-trial evidence, the results should be generalised cautiously: no single comparison can represent every well-formulated vegan diet or every Mediterranean diet, and the durations (8–16 weeks) preclude inference about long-term durability. In quantitative terms, the vegan arm of the Landry twin trial showed an additional LDL cholesterol reduction of approximately 13.9 mg/dL (about 0.36 mmol/L), a fasting insulin reduction of roughly 2.9 uIU/mL, and a mean body weight reduction about 1.9 kg greater than the omnivorous arm over 8 weeks, illustrating the direction and approximate magnitude of short-term biomarker change while leaving long-term clinical significance unresolved.

A frequent concern about strict plant-based dietary patterns is anabolic adequacy. Two recent contributions address this concern directly. Domić et al. [16] reported in a randomised controlled crossover trial in 34 community-dwelling older adults—with 10 days per diet period under fully controlled isocaloric and isonitrogenous conditions and 60% animal-source protein in the omnivorous arm—that a well-balanced vegan diet did not compromise daily mixed muscle protein synthesis rates relative to the omnivorous diet. López-Moreno et al. [17] showed in a systematic review and meta-analysis of randomised controlled trials that plant-based dietary patterns did not impair muscular strength compared with omnivorous patterns. Together, these findings qualify earlier mechanistic concerns about plant-based protein anabolic responses and indicate that, when total protein intake and amino acid quality are appropriately planned, vegan dietary patterns can support muscle outcomes equivalent to those of omnivorous patterns. Mechanistic studies remain consistent with this conclusion: a single dose of plant protein produces a smaller acute anabolic stimulus than an equivalent dose of high-quality animal protein, but the gap is attenuated when total protein dose, leucine content, and meal timing are appropriately managed [37,38]. A separate controlled trial reported greater absorption of non-haem iron in long-term vegans than in omnivores under matched conditions [14], a finding that qualifies any mechanistic prediction that lower theoretical bioavailability of plant-source iron necessarily translates into worse iron status. Possible mediators include adaptive regulation of hepcidin (the principal hormonal regulator of systemic iron homeostasis) in response to body iron demand, up-regulation of intestinal divalent metal transporter 1 (DMT1) expression in vegan participants with lower ferritin, and chronic exposure to higher dietary vitamin C from plant-rich diets [39]. Bioavailability should therefore be evaluated as a context-dependent biological process rather than as a fixed multiplicative penalty applied to plant-source intake.

The integrative reading of this evidence is conservative but constructive. When vegan and vegetarian dietary patterns are well designed, minimally processed, and nutritionally planned, they can show cardiometabolic advantages in the short term even relative to healthy omnivorous comparators (OMNIVEG, Landry, Barnard); when compared with the Mediterranean pattern, they may be approximately equivalent on a net cardiometabolic basis with differential effects across specific markers (CARDIVEG). Concerns about impaired muscle protein synthesis or muscular strength are not confirmed by the available short-term matched evidence when total protein intake and amino acid quality are adequately planned (Domić, López-Moreno meta-analysis). The evidence, however, remains short-term and dominated by intermediate-biomarker endpoints; long-term randomised trials directly comparing well-planned vegan and high-quality omnivorous patterns on hard clinical endpoints—cardiovascular events, fractures, sarcopenia, cancer incidence, cognitive decline, mortality—remain absent, and the most defensible reading of the existing evidence supports well-formulated plant-rich patterns rather than refuting them, without establishing universal hierarchy.

Table 2 collates the most informative recent randomised controlled trials that have directly compared well-formulated plant-based patterns against high-quality omnivorous comparators. OMNIVEG, Landry, CARDIVEG, Barnard, and Domić share an important methodological feature: their comparator is a high-quality Mediterranean or omnivorous diet, not the modern Western diet. Their results are convergent rather than uniform: well-planned vegan and vegetarian patterns can improve selected cardiometabolic markers relative to high-quality omnivorous comparators (LDL-C in OMNIVEG, Landry, Barnard, CARDIVEG; body weight in Landry and Barnard), without compromising muscle protein synthesis or exercise metabolism (Domić, OMNIVEG 2025). The principal interpretive caveats are the short durations (10 days to 16 weeks per arm), the small sample sizes, and the reliance on intermediate biomarkers rather than hard clinical endpoints—features that mark this body of work as an important advance over earlier comparisons against Western diets but still insufficient for long-term clinical claims.

## 5. Methodological Pitfalls and Interpretive Constraints

The following considerations are not a wholesale critique of plant-based dietary patterns, which are well supported, but a set of constraints on overly strong or overly weak claims about animal-food exclusion. They should be read symmetrically: each applies to vegan, vegetarian, and omnivorous dietary patterns alike. The single most influential interpretive limitation is the routine comparison of one dietary identity against a comparator whose composition is poorly characterised. Demonstrating that a minimally processed plant-rich pattern is superior to a Western diet is a low evidentiary bar; demonstrating that strict animal-food exclusion adds value beyond what is achieved by a similarly well-formulated omnivorous diet is a more demanding test that recent randomised work—OMNIVEG, Landry, Barnard, and Domić—has begun to address, with results that support well-formulated plant-based patterns rather than refute them. Within-family or genetically informed designs can partially mitigate residual confounding but remain rare in this literature; the Landry identical-twin trial [27] is a notable example, and Mendelian randomisation studies of habitual meat or fish intake have begun to address residual confounding for selected nutritional exposures, although instrument validity and pleiotropy remain analytic challenges [40].

Individuals who voluntarily adopt vegetarian or vegan diets in high-income settings differ from non-vegetarians in multiple behaviours that themselves influence chronic disease risk—lower smoking prevalence, lower alcohol intake, higher physical activity, greater supplement use, higher educational attainment, and greater engagement with preventive healthcare [4,5,11]. Statistical adjustment reduces but does not eliminate this confounding. Most large vegetarian cohort analyses additionally rely on self-reported food frequency questionnaires, which are useful for ranking populations but vulnerable to recall bias, social desirability bias, exposure misclassification, and limited resolution of food quality, food processing, and cooking methods [41]. FFQs are particularly insensitive to the food processing dimension: a participant consuming predominantly ultra-processed plant products and one consuming a whole-food plant-based diet may be classified identically despite radically different physiological exposures [8,9,10]. Repeated 24 h recalls, weighed food records, and nutritional biomarkers (urinary nitrogen, sucrose, potassium, plasma carotenoids; recovery and concentration biomarkers) reduce but do not eliminate measurement error, and few cohorts of vegan participants have implemented these designs at scale [42].

Most vegan and vegetarian dietary trials use intermediate biomarkers as primary outcomes—LDL cholesterol, fasting insulin, body weight, blood pressure, and inflammatory markers—which are valid surrogates but do not establish superiority for clinically meaningful endpoints. A hierarchy of outcomes, from mechanistic markers to surrogate biomarkers to hard clinical endpoints, should therefore be made explicit in evidence summaries and clinical guidance, and PREDIMED and CORDIOPREV [21,22] remain the principal examples of dietary pattern trials with hard cardiovascular endpoints—both involving Mediterranean comparators rather than vegan arms. Most randomised trials of vegan dietary patterns also extend across weeks to months; long-term randomised trials directly comparing a well-planned vegan diet with a high-quality omnivorous diet, matched for energy intake, plant food intake, and lifestyle support, remain absent. Until such trials are completed, conclusions about the long-term superiority of any pattern are necessarily provisional, particularly for outcomes that develop over decades such as fracture risk, cancer incidence, sarcopenia, and cognitive decline. Finally, the terms “plant-based” and “vegan” mask substantial heterogeneity, as do “omnivorous” and “Mediterranean”: stratifying by NOVA processing categories and by hPDI/uPDI score reveals that ultra-processed plant-based exposures behave differently from whole-food plant-based exposures [7,8,9,10], and the same logic applies symmetrically to omnivorous diets. The unavoidable implication is that “vegan” and “omnivorous” are insufficient as primary exposure variables; pattern quality, processing level, and nutrient adequacy carry most of the explanatory weight. Where meta-analyses of plant-based dietary patterns report statistical heterogeneity, it is frequently high (I-squared often exceeding 50–75%), consistent with variation in comparator quality, processing level, and adherence; this heterogeneity is itself an argument for stratified rather than pooled interpretation and constrains the derivation of a single summary effect size. A related interpretive caution concerns how processing was operationalised: large cohorts such as the UK Biobank classified foods into NOVA categories by mapping brief touchscreen questionnaires and a limited number of 24 h dietary recalls onto processing groups, an imperfect approximation that can misclassify ultra-processed exposure and that should temper confidence in NOVA-based cohort signals.

Table 3 organises the principal methodological considerations that condition the interpretation of plant-based diet research and articulates a recommended response for each. Read horizontally, the table makes explicit the symmetry that this review has argued for throughout: comparator quality, healthy-user bias, dietary misclassification, intermediate-versus-hard endpoints, trial duration, within-pattern heterogeneity, and conflict-of-interest disclosure apply equally to vegan, vegetarian, and omnivorous comparisons. The recommended methodological responses point towards a research agenda that prioritises within-twin or within-sibling designs, repeated 24 h recalls and metabolomic dietary signatures, pre-specified hard endpoints, longer follow-up, and stratification by NOVA processing and hPDI/uPDI on both sides of the comparison.

## 6. Food Matrix Biology and Nutrients Requiring Attention

**Figure 1:**
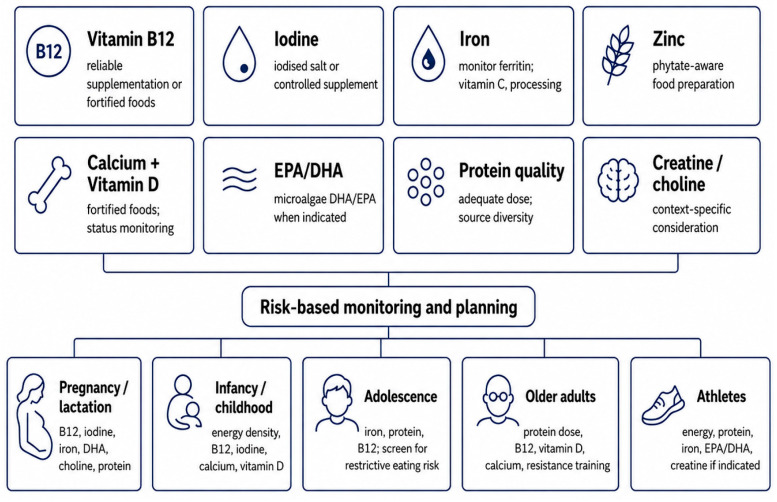
Nutrients requiring systematic attention in vegan dietary patterns across life stages and physiological contexts. Nutrients are ordered by supplementation priority: vitamin B12 is flagged as a non-negotiable, mandatory supplement in all vegan diets (highest-priority marker), whereas iron, zinc, calcium, iodine, EPA/DHA, and vitamin D are conditional priorities that can frequently be addressed through fortified foods, food-based strategies, or targeted supplementation according to life stage and physiological context.

Bioavailability modifiers in plant foods—formerly termed “antinutrients”—are biologically active compounds whose effect on nutrient absorption depends on dose, food matrix, processing, gut function, and physiological demand. Neither dismissing these compounds nor framing plant foods as inherently toxic reflects current evidence. Phytate (myo-inositol hexaphosphate), the principal storage form of phosphorus in cereals, legumes, nuts, and seeds, chelates divalent cations (notably zinc, iron, and calcium) and reduces their absorption in proportion to the phytate-to-mineral molar ratio [13]; soaking, sprouting, fermentation (including sourdough leavening), and adequate cooking lower phytate content and improve mineral bioavailability [43]. Phytate-to-iron molar ratios above ~1 and phytate-to-zinc ratios above ~15 are commonly used thresholds for poor mineral bioavailability in plant-based dietary contexts [13], and the clinical relevance is greater in populations whose diet is dominated by unrefined cereals and legumes without traditional processing, in growing children, and in iron- or zinc-deficient adults. Oxalates form insoluble complexes with calcium and reduce its absorption; their clinical consequence is more relevant for kidney stone risk in susceptible individuals than for population-level calcium adequacy. Tannins in tea, coffee, cocoa, and red wine inhibit non-haem iron absorption when consumed concurrently with iron-containing meals, and the practical recommendation is to separate iron-rich meals from tannin-rich beverages in populations at risk of iron deficiency. Lectins and protease inhibitors in raw legumes reduce protein digestibility but are denatured by adequate cooking; goitrogenic compounds in cruciferous vegetables, soy isoflavones, and millet may interfere with iodine utilisation only in individuals with marginal iodine intake. Crucially, plant-food matrices also contribute polyphenols, isoflavones, and sulphur compounds with positive effects on glycaemic control, antioxidant defence, and gut microbiota modulation; polyphenol metabolism by gut microbiota generates urolithins, equol, and short-chain fatty acids that modulate inflammation, insulin signalling, and endothelial function [44]. Recent controlled-trial evidence further suggests adaptive regulation of non-haem iron absorption in long-term vegans, indicating that lower theoretical bioavailability does not necessarily translate into worse iron status [14]. Figure 1 summarises the principal nutrients requiring systematic attention across life stages and physiological contexts. Modern industrial processing of plant-based meat analogues adds a further consideration to this traditional-processing discussion: high-moisture extrusion, texturisation, and the use of purified protein isolates reshape the food matrix in ways that can either enhance mineral bioavailability (through phytate reduction and deliberate fortification with iron and zinc) or impair it (through added calcium, residual phytate carried in isolates, or the formation of poorly absorbed mineral complexes); so, the net effect of analogues on iron and zinc absorption is product-specific and cannot be assumed favourable without compositional and bioavailability data.

A defined set of nutrients requires systematic attention in vegan diets, and fortification or supplementation is a normal tool of contemporary public health nutrition rather than a marker of dietary inferiority. Vitamin B12 is the clearest example: unfortified plant foods do not provide a reliable source, and serum cobalamin deficiency is common in vegans not using supplements or fortified foods [12,45]. Subclinical deficiency, detectable through elevated methylmalonic acid and homocysteine, can persist for years before overt manifestations appear, and is particularly consequential during pregnancy, lactation, infancy, and old age; both the updated Academy of Nutrition and Dietetics position [1] and the German Nutrition Society 2024 update [3] make reliable B12 supplementation or fortification a non-negotiable feature of vegan dietary patterns. Pharmacokinetic data indicate that daily oral cyanocobalamin doses of 25–100 µg, weekly doses of 2000 µg, or—where adherence is uncertain—parenteral hydroxocobalamin can reliably restore status in most patients [46]. Plant-source iron is exclusively non-haem iron whose absorption is markedly inhibited by phytates, polyphenols, and calcium and enhanced by ascorbic acid [13]; vegans frequently meet the recommended iron intake on paper while showing lower ferritin in some studies, particularly in menstruating women, endurance athletes, children during rapid growth, and pregnant women [11]. Zinc absorption is inhibited by phytates and is lower from plant-source than animal-source foods, with a systematic review and meta-analysis reporting lower mean serum zinc and lower estimated zinc intake in vegetarian compared with omnivorous diets [47]. Iodine deficiency is a risk in vegan diets without iodised salt, controlled seaweed intake, or specific supplementation [3,11], and iodised salt or a defined low-dose iodine supplement (typically 150 µg/day in adults, 220–290 µg/day during pregnancy and lactation) is recommended. For practical purposes, these nutrients can be stratified by supplementation priority into a non-negotiable tier (vitamin B12, mandatory in every vegan diet) and a conditional tier (iron, zinc, iodine, calcium, vitamin D, and EPA/DHA) whose supplementation depends on intake, life stage, biochemical monitoring, and physiological demand rather than being universally obligatory.

Calcium and vitamin D status are relevant to bone mineral density, fracture risk, muscle function, and ageing. EPIC-Oxford reported higher rates of total and site-specific fractures, particularly hip fractures, in vegans than in meat eaters, with the association partly explained by lower BMI, lower calcium intake, and lower protein intake [36]; fortified plant beverages provide calcium and vitamin D in many high-income settings, but fortification levels are variable and dietary calcium is often obtained from sources with low bioavailability or high oxalate content, making active monitoring appropriate in long-term vegan diets, particularly in postmenopausal women, adolescents during peak bone mass accrual, and patients with malabsorption. Vegan diets contain α-linolenic acid but its conversion to long-chain omega-3 fatty acids (EPA and especially DHA) is limited and variable; functional DHA requirements during pregnancy, lactation, infancy, and early childhood are not reliably met by ALA alone, and microalgae-derived DHA supplementation is the preferred strategy in strict vegan diets [48,49]. Plant proteins generally have lower digestibility and a less complete indispensable amino acid profile (notably lower leucine and lysine content) than most animal proteins, translating into a lower DIAAS [15]; the acute anabolic response to a single plant protein meal is typically lower than to a matched dose of high-quality animal protein, although this gap can be attenuated by increasing total protein dose, combining complementary plant sources, or selecting protein isolates with favourable amino acid profiles [37]. As discussed above, recent randomised work shows that this acute mechanistic gap does not translate into impaired daily mixed muscle protein synthesis or muscular strength when total protein and amino acid quality are appropriately planned [16,17]. Conditionally relevant compounds—creatine, carnosine, taurine, retinol, and choline—are present at low or negligible concentrations in vegan diets; creatine supplementation may be considered in athletic, ageing, or cognitive performance contexts, and choline requirements increase during pregnancy and lactation and are often unmet in vegan women without conscious planning, with potential implications for foetal neurodevelopment [50].

Table 4 synthesises the nutrients requiring systematic attention in vegan and vegetarian diets together with their principal sources, supplementation/fortification rationale, and clinical monitoring strategy. Two organising principles emerge. First, the nutrients of concern are not uniformly equivalent: vitamin B12 is the only nutrient for which planned supplementation or fortification is genuinely non-negotiable in strict vegan diets, whereas iron, zinc, calcium, vitamin D, iodine, EPA/DHA, and protein quality require contextual monitoring that depends on age, sex, physiological state, and baseline status. Second, the conditionally relevant compounds (creatine, carnosine, taurine, choline) are not classical essential nutrients but warrant attention in specific populations—athletes, older adults, pregnant and lactating women—rather than as universal supplementation requirements for every vegan adult.

## 7. Human Dietary Flexibility and the Quality-over-Identity Argument

Human dietary evolution is best characterised by ecological flexibility rather than by a single ancestral pattern [18,19]. Archaeological, isotopic, and dental microwear evidence document substantial variation in ancestral human diets across geography, climate, season, and technology: coastal populations incorporated extensive marine foods, tundra populations relied heavily on animal foods including marrow and organs, and tropical forest populations made greater use of fruits, tubers, honey, and seasonally available animal protein. The unifying feature is dietary flexibility rather than any single dietary template, and attempts to reconstruct one “Palaeolithic diet” misrepresent this diversity. Animal-source foods historically provided concentrated high-quality protein, vitamin B12, retinol, haem iron, zinc, long-chain omega-3 fatty acids, and conditionally relevant compounds such as taurine, creatine, and carnosine [20]; cooked tubers, fruits, seeds, nuts, honey, and fermented plant foods have been equally central to most ancestral diets. The evolutionary argument here is therefore contextual and explicitly non-prescriptive: it supports the feasibility and historical recurrence of multiple dietary configurations but does not demonstrate that any contemporary dietary pattern—vegan, vegetarian, or omnivorous—is biologically superior in modern societies that have fortification, supplementation, year-round food availability, and detailed nutritional knowledge. Strict veganism is feasible in contemporary societies precisely because of vitamin B12 supplementation, food fortification, industrial logistics, and contemporary nutritional knowledge, and the relevant question for contemporary recommendation is not what was eaten in the past but which patterns deliver favourable outcomes today.

The central proposition that emerges from this synthesis is that dietary pattern quality, food processing, nutrient adequacy, and adherence—applied symmetrically to plant-based and omnivorous patterns—explain more variance in long-term outcomes than the binary inclusion or exclusion of animal-source foods. The Mediterranean dietary pattern is the most extensively studied omnivorous pattern with hard-outcome trial evidence: PREDIMED demonstrated that a Mediterranean diet supplemented with extra-virgin olive oil or nuts reduced major cardiovascular events in primary prevention [21], and CORDIOPREV extended this finding in secondary prevention [22]; neither trial adjudicates the vegan-versus-omnivorous comparison directly because no vegan arm was included. Within plant-rich comparisons, CARDIVEG [23], Barnard 2022 [24], and OMNIVEG [25,26] show that vegan or vegetarian patterns can match or exceed Mediterranean omnivorous patterns on selected intermediate biomarkers; long-term hard-outcome trials directly comparing well-planned vegan and high-quality omnivorous patterns remain absent. In all comparators considered, food processing and overall pattern quality dominate the variance more than the binary inclusion or exclusion of any single food group. The constructive conclusion is therefore not that any pattern is universally superior, but that well-formulated plant-rich patterns—vegan, vegetarian, Mediterranean omnivorous, and minimally processed omnivorous—are all defensible options, and that the public health priority is shifting populations towards minimally processed, plant-rich patterns regardless of label.

## 8. Clinical Application Across Life Stages and Special Populations

The clinical interpretation of vegan and vegetarian diets cannot be uniform across life stages and physiological states; recommendations that are sound for healthy adults may be inadequate or unsafe in populations with increased nutritional demand. Pregnancy and lactation entail elevated requirements for vitamin B12, iodine, iron, choline, DHA, and adequate high-quality protein [48,52]: well-planned vegan pregnancies, supported by reliable B12 and DHA supplementation, iodine adequacy, iron monitoring, and adequate protein intake, can support maternal and foetal nutritional adequacy when appropriately supervised [48], whereas inadequately planned vegan pregnancies, particularly those without B12 supplementation, are associated with measurable risks of maternal and infant deficiency [45]. Position statements [1,3] recommend individualised counselling and structured supplementation rather than blanket endorsement or rejection, and recent cohort data suggest that pregnant women adhering to vegan diets without structured nutritional counselling may be at increased risk of low birth weight and small-for-gestational-age outcomes—an observation that underscores the importance of prenatal dietetic supervision rather than the inadequacy of the dietary pattern per se [53]. Children, with their high energy and nutrient density requirements relative to gastric capacity, require deliberate planning, supplementation, and clinical monitoring on vegan diets; the Academy of Nutrition and Dietetics considers planned vegetarian and vegan diets appropriate for all stages of life conditional on adequate planning [1], whereas the DGE 2024 statement is more conservative for early childhood and recommends specialised paediatric supervision [3]. Both positions are compatible: when appropriately supervised, vegan diets are feasible; without adequate planning, supplementation, and monitoring, the risk of clinically relevant nutrient inadequacy is higher. Adolescent growth, particularly in female adolescents, increases iron and protein requirements [52], and in susceptible adolescents, restrictive dietary identities—including but not limited to veganism—may sometimes overlap with or mask disordered eating behaviours, so clinical assessment should distinguish ethically motivated, well-planned vegan adoption from restrictive eating with food group elimination as a control strategy.

Sarcopenia prevention in older adults requires sustained adequate protein intake, frequently in the range of 1.0–1.2 g/kg/day or higher, with attention to per-meal protein distribution and leucine-rich protein sources [51,54]; recent randomised evidence indicates that a well-balanced vegan diet matched for energy and nitrogen does not compromise daily mixed muscle protein synthesis in active older adults [16] and that plant-based dietary patterns do not impair muscular strength in randomised trials [17]; so, vegan older adults can meet anabolic demands when total protein and amino acid quality are planned, often through larger total protein volumes or strategic use of plant protein isolates, while vitamin D, calcium, B12, and resistance training remain pertinent. In athletes, who have elevated requirements for protein, iron, energy availability, and selected conditionally essential compounds such as creatine, the OMNIVEG trials demonstrate that transitioning from a traditional to a vegan Mediterranean diet did not adversely affect cardiorespiratory or substrate utilisation responses to progressive exercise in healthy active men [25,26], and the López-Moreno meta-analysis indicates no impairment of muscular strength in randomised trials of plant-based patterns [17]; vegan athletes can therefore compete and adapt at high levels with attention to total protein, high-quality protein sources or isolates, iron status, EPA/DHA, and context-specific use of creatine supplementation where appropriate, with planning rather than exclusion as the operative variable. In patients with gastrointestinal disorders, high-fibre, high-FODMAP (fermentable oligo-, di-, mono-saccharides and polyols), or legume-rich diets may exacerbate symptoms in irritable bowel syndrome, inflammatory bowel disease in flare, and some functional gastrointestinal disorders (tolerance is individual and should guide rather than override dietary identity); in patients with malabsorption (coeliac disease, post-surgical anatomy), micronutrient surveillance is particularly important for vegan diets. In patients with current or historical eating disorders, the adoption of any restrictive dietary pattern, including veganism, warrants careful multidisciplinary assessment: restrictive dietary identities can sometimes serve as a socially acceptable cover for disordered eating, a clinical reality that should inform individualised assessment rather than becoming a generalised critique of veganism.

Translating this evidence into routine clinical practice requires a risk-based monitoring framework rather than universal intensive surveillance. For individual patients adopting vegan or vegetarian patterns, clinicians should consider risk-based monitoring of vitamin B12 status (serum cobalamin, methylmalonic acid, homocysteine), ferritin and transferrin saturation, vitamin D status, calcium intake, iodine intake or thyroid function where appropriate, omega-3 index where available, lipid profile, bone health indicators when clinically indicated, and protein adequacy. Reliable B12 supplementation should be considered non-negotiable in strict vegan diets, framed as a normal and tractable element of contemporary nutritional practice rather than as evidence of dietary failure. Public health guidance, by contrast, should resist presenting any single dietary identity—vegan, vegetarian, or omnivorous—as inherently or universally superior, given the methodological limitations summarised above and the absence of long-term randomised evidence directly comparing well-planned vegan with high-quality omnivorous patterns; a more defensible message centres on food quality, food processing, nutrient density, adherence, and individual physiological context. Adherence and access further condition real-world effectiveness. A well-planned vegan diet demands a comparatively high level of nutritional literacy, deliberate meal planning, and consistent supplementation, which can be harder to sustain in the general population than more familiar patterns such as the Mediterranean diet; and the cost and availability of nutrient-dense plant foods, fortified products, and supplements can represent a meaningful barrier for lower-income and food-insecure households. These implementation barriers, together with the elevated needs of vulnerable groups such as children, pregnant and lactating women, and older adults, should be weighed explicitly when translating dietary pattern evidence into individual advice and public health policy.

## 9. A Pattern Quality Framework for Comparing Dietary Patterns

Five archetypal dietary patterns can be located in the evidence, as summarised in Table 5. The ultra-processed Western omnivorous diet—high in processed meat, refined grains, added sugars, alcohol, and industrial fats—is consistently associated with adverse cardiometabolic outcomes. The poorly planned vegan diet, with heavy reliance on refined grains, sweetened plant beverages, plant-based meat analogues high in saturated fat and sodium, and limited supplementation, can produce a cardiometabolic risk profile that is similar to or worse than the ultra-processed Western omnivorous diet, illustrating that the “vegan” label is insufficient by itself to confer cardiometabolic benefit. The well-planned vegan diet—minimally processed plant foods, fortified foods, reliable B12 and DHA supplementation, monitored iron and zinc—is cardiometabolically favourable and, in recent direct comparisons, capable of producing additional improvements in selected biomarkers relative to high-quality omnivorous comparators. The Mediterranean omnivorous diet retains hard-outcome trial evidence (PREDIMED, CORDIOPREV) and is plant-rich, minimally processed, and includes fish, olive oil, eggs, dairy, and limited red and processed meat. The minimally processed omnivorous diet—plant-rich, minimally processed, nutrient-dense, with strategic inclusion of fish, eggs, dairy where tolerated, and limited unprocessed meat—is plausibly favourable when well executed, although with less direct hard-outcome RCT evidence than the Mediterranean pattern.

Table 5 maps five archetypal dietary patterns—ultra-processed Western omnivorous, poorly planned vegan, well-planned vegan, Mediterranean omnivorous, and minimally processed omnivorous—against plant-food density, processing level, animal-food inclusion, likely cardiometabolic profile, principal nutrients requiring planning, and supplementation need. The principal lesson from the table is the symmetry of risk: the ultra-processed Western omnivorous pattern and the poorly planned vegan pattern occupy adjacent zones of an adverse cardiometabolic profile, while the well-planned vegan, the Mediterranean omnivorous, and the minimally processed omnivorous patterns occupy adjacent zones of a favourable profile. Animal-food inclusion is therefore a weaker determinant of long-term outcomes than the conjunction of pattern quality, processing level, and nutrient adequacy.

Within this framework, the principal axis of public health relevance is dietary pattern quality, not animal-food inclusion: the well-planned vegan, the Mediterranean omnivorous, and the well-formulated minimally processed omnivorous patterns can all support long-term health, while the ultra-processed Western omnivorous and the poorly planned vegan patterns are both likely to produce adverse cardiometabolic outcomes. This reframing avoids the false dichotomy that has dominated public nutritional discourse and aligns clinical and public health messaging with the most defensible reading of contemporary evidence: prioritise food quality, processing level, nutrient density, adherence, and outcome hierarchy over binary dietary identities. Current evidence accordingly supports shifting population diets towards predominantly plant-based, minimally processed patterns rich in legumes, fruits, vegetables, whole grains, nuts, and seeds, with a clear reduction in processed meat and context-appropriate reductions in red meat and ultra-processed foods. Well-planned vegan and vegetarian diets are valid and potentially advantageous expressions of this broader pattern, although they require systematic attention to specific nutrients of concern. The relevant comparison is not idealised veganism versus the Western diet, nor poorly planned veganism versus idealised omnivory, but well-formulated dietary patterns compared under equivalent conditions of dietary support and nutrient adequacy.

Table 6 provides an evidence map by outcome domain, listing the principal studies, the direction of effect, and a narrative certainty appraisal that combines consistency, study design, comparator quality, follow-up duration, and the distinction between hard endpoints and intermediate biomarkers. The map clarifies where the evidence is strongest and where it is most contingent. Mediterranean hard-endpoint trials anchor the highest-certainty signal for cardiovascular events; randomised trials with healthy omnivorous comparators provide moderate-to-high certainty for short-term LDL cholesterol and body weight effects of well-planned plant-rich patterns; cancer incidence and bone outcomes remain at low-to-moderate certainty owing to residual confounding and the predominance of observational evidence; and exercise metabolism and muscular outcomes show no adverse signal in short-term matched comparisons but lack long-term confirmation. The cumulative reading is consistent with the synthesis above: well-formulated plant-rich patterns are well supported for cardiometabolic risk markers, but universal claims of long-term clinical superiority require evidence that has not yet been generated.

## 10. Limitations, Research Gaps, and Future Directions

This review has several limitations that condition its conclusions. Evolutionary and anthropological evidence is necessarily indirect and is therefore presented as context rather than as a basis for hierarchy among contemporary patterns. Dietary patterns are complex exposures that cannot be reduced to the presence or absence of isolated food groups; the health effects of any pattern depend on the overall food matrix, the degree of processing, energy intake, protein quality, micronutrient density, fibre intake, fatty acid profile, supplementation practices, cooking methods, and long-term adherence. Vegan, vegetarian, and omnivorous diets differ widely in quality across populations, and this heterogeneity limits the extent to which broad dietary labels can be used to infer biological or clinical superiority. Long-term randomised controlled trials directly comparing well-planned vegan diets with high-quality omnivorous diets remain scarce, and much of the current evidence comes from observational cohorts, short-term trials, and studies using intermediate cardiometabolic markers. The review focused primarily on human biology, nutritional adequacy, clinical outcomes, and methodological interpretation; environmental, ethical, cultural, religious, and economic considerations may legitimately influence dietary choices at the individual and societal levels but are not equivalent to evidence of biological superiority of any pattern.

Future research should move beyond broad comparisons between vegan or vegetarian diets and poorly characterised omnivorous diets. The most important priority is the design of long-term randomised controlled trials directly comparing well-planned vegan diets with high-quality omnivorous dietary patterns, matched or carefully balanced for overall dietary quality, processing level, energy intake, fibre content, dietary support intensity, and health-conscious behavioural support. A second priority is the incorporation of objective biomarkers of dietary intake, nutrient status, and physiological adaptation—methylmalonic acid, homocysteine, ferritin, transferrin saturation, zinc, iodine, vitamin D, omega-3 index, calcium metabolism, nitrogen balance, amino acid availability, and metabolomic dietary signatures. A third priority is the prioritisation of hard clinical and functional outcomes—fractures, bone mineral density trajectories, sarcopenia, muscle strength, physical performance, cardiovascular events, stroke subtypes, cognitive decline, fertility, pregnancy outcomes, immune function, frailty, hospitalisation, and all-cause mortality—rather than reliance on intermediate biomarkers alone. Special populations (pregnancy, infancy, adolescence, ageing, athletes, patients with chronic disease) require dedicated investigation, and whole-food plant-based, ultra-processed plant-based, Mediterranean, and minimally processed omnivorous diets should be evaluated as distinct exposures rather than aggregated under coarse labels. Further work is needed on protein quality and anabolic response in older adults and athletes, on long-term iron and bone outcomes in vegan populations, and on the public health translation of pattern quality frameworks into policy and clinical practice. For this purpose a high-quality omnivorous comparator should be defined explicitly rather than by default—operationally, a minimally processed, plant-rich omnivorous pattern (for example, a Mediterranean-type diet) matched to the plant-based arm for energy intake, fibre, degree of processing (NOVA), and behavioural and dietetic support—so that future trials contrast well-formulated patterns on equal terms rather than comparing an idealised vegan diet with a low-quality Western diet, a recurrent error in the older literature.

## 11. Conclusions

Current evidence supports minimally processed, plant-rich dietary patterns as favourable for cardiometabolic health, whether vegan, vegetarian, or omnivorous. Well-planned vegan and vegetarian diets can improve LDL cholesterol, body weight, insulin sensitivity, and several cardiometabolic risk markers in short-term trials, including some using healthy omnivorous comparators. The evidence nonetheless remains limited by short follow-up, reliance on intermediate biomarkers, heterogeneity in plant-based diet quality, and the scarcity of long-term randomised trials with hard clinical outcomes; claims of universal biological superiority of strict animal-food exclusion are therefore premature. Equally, the existing evidence does not justify portraying high-quality omnivorous diets as biologically superior to well-formulated whole-food plant-based patterns. The most defensible synthesis is that dietary pattern quality, food processing level, nutrient adequacy, and adherence explain more variance in long-term outcomes than the categorical presence or absence of animal-source foods, and that well-formulated plant-rich patterns—vegan, vegetarian, Mediterranean omnivorous, or minimally processed omnivorous—are all defensible options under equivalent conditions of dietary support. Public health messaging should accordingly prioritise dietary quality, minimally processed plant foods, reduced processed meat and context-appropriate reductions in red meat, nutrient adequacy, adherence, outcome hierarchy, and lower ultra-processed food intake rather than binary dietary identities. It must be emphasised that, to date, no long-term randomised controlled trial has compared a well-planned vegan diet with a high-quality omnivorous diet using hard clinical endpoints such as mortality, myocardial infarction, stroke, or fractures; the observed advantages of plant-based patterns rest largely on intermediate biomarkers such as LDL cholesterol and fasting insulin, which are valid surrogates but do not by themselves guarantee long-term clinical superiority. Conclusions about the comparative long-term benefit of any well-formulated dietary pattern therefore remain provisional until such trials are conducted.

## Figures and Tables

**Table 1:** Principal prospective cohort studies of vegetarian, vegan, and plant-based dietary patterns: design and headline findings.

Cohort (Ref.)	Country/Setting	*n* (Veg/Vegan Subset)	Follow-Up	Exposure Assessment	Main Findings (Veg/Vegan vs. Omnivore)	Limitations	Methodological Quality/Confidence
Adventist Health Study-2 [5]	USA and Canada (Seventh-day Adventists)	~96,000 total; ~28,000 vegetarian incl. ~5500 vegan	5.8 yr median	FFQ at baseline	Lower all-cause mortality in vegetarians (HR ≈ 0.88) and some cause-specific reductions.	Selected religious cohort; possible residual confounding.	Moderate–high (large prospective cohort, validated FFQ; limited by selected religious population and residual confounding)
EPIC-Oxford [6,36]	United Kingdom	~48,000 total; ~7400 vegetarian, ~2500 vegan	18 yr	FFQ at baseline	Lower IHD in fish eaters and vegetarians; higher total and haemorrhagic stroke in vegetarians; higher fracture risk in vegans (HR ≈ 1.4; hip HR ≈ 2.3).	Healthy-volunteer bias; baseline-only intake.	Moderate (large sample, long 18-year follow-up; weakened by baseline-only FFQ and healthy-volunteer bias)
NutriNet-Santé [10]	France	~70,000 incl. ~2400 vegetarian/vegan	7 yr median	Repeated 24-h recall	Plant-based + low ultra-processed pattern associated with lower CVD risk; balance of animal/plant origin matters.	Web-based self-recruitment; attrition.	Moderate (repeated 24-h recalls strengthen exposure capture; limited by web self-recruitment and attrition)
UK Biobank—hPDI/uPDI [8]	United Kingdom	~126,000	~12 yr	Touchscreen + 24-h recalls	Higher hPDI associated with lower mortality, CVD and cancer; higher uPDI associated with opposite signal.	Coarse dietary assessment; pattern variation over time.	Moderate (very large sample; limited by coarse dietary assessment and pattern drift over time)
UK Biobank—NOVA [9]	United Kingdom	~118,000	~12 yr	Touchscreen + 24-h recalls	Ultra-processed plant-based foods adverse; whole-food plant-based favourable.	NOVA classification disputed; residual confounding.	Low–moderate (very large sample, but NOVA operationalisation from imperfect survey data is disputed and prone to residual confounding)

**Table 2:** Selected randomised controlled trials directly comparing vegan or vegetarian dietary patterns against high-quality omnivorous (Mediterranean) comparators.

Trial (Ref.)	Design	*n*	Duration	Intervention	Comparator	Primary Outcome(s)	Main Result
Landry et al. 2023 [27]	Randomised parallel in identical twins	22 pairs (44)	8 wk	Healthy vegan diet	Healthy omnivorous diet	LDL-C, fasting insulin, body weight	Greater LDL-C, fasting insulin, and body weight reductions in vegan arm.
OMNIVEG 2024 [25]	Controlled crossover dietary intervention	14 healthy physically active men	3 wk traditional Mediterranean diet; 1 wk washout; 4 wk vegan Mediterranean diet	Vegan Mediterranean diet	Traditional Mediterranean diet	Total cholesterol, LDL-C	Reduced total cholesterol and LDL-C after transition to vegan Mediterranean diet.
OMNIVEG 2025 [26]	Controlled crossover; progressive exercise	as above	as above	Vegan Mediterranean diet	Traditional Mediterranean diet	Fat oxidation, Fatmax, EE, HR, RPE during exercise	No deterioration in any exercise metabolic or perceptual outcome after transition.
Domić et al. 2025 [16]	Randomised controlled crossover	34 community-dwelling older adults	10 d per diet period	Controlled well-balanced vegan diet	Controlled omnivorous diet (isocaloric/isonitrogenous; 60% animal protein)	Daily mixed muscle protein synthesis	No difference between vegan and omnivorous diets in daily mixed muscle protein synthesis.
Barnard et al. 2022 [24]	Randomised crossover	62	16 wk per arm	Low-fat vegan diet	Mediterranean diet	Body weight, lipids, insulin resistance, BP	Vegan: greater weight, lipid, and insulin resistance reductions; Mediterranean: better BP.
CARDIVEG 2018 [23]	Randomised crossover, hypocaloric	~110	3 mo × 2	Lacto-ovo-vegetarian hypocaloric	Mediterranean hypocaloric	Weight, lipids, glucose, oxidative stress	Equivalent weight loss; vegetarian: greater LDL-C reduction; Mediterranean: greater triglyceride reduction.

**Table 3:** Methodological considerations affecting interpretation of plant-based-diet research, applied symmetrically to vegan, vegetarian, and omnivorous patterns.

Consideration	Concrete Manifestation	Consequence	Methodological Safeguards
Comparator validity	Vegan/vegetarian arm compared with usual Western diet rather than high-quality omnivorous diet.	Apparent superiority may reflect comparator inferiority.	Use Mediterranean or minimally processed omnivorous comparator (Landry, OMNIVEG, CARDIVEG, Barnard, Domić).
Healthy-user bias	Vegans show lower smoking, lower alcohol, higher exercise, more supplements, higher education.	Residual confounding inflates apparent benefit.	Within-twin or within-sibling designs; quantitative bias analysis; pre-registration.
Self-reported dietary assessment	FFQs cannot discriminate ultra-processed plant products from whole-food plant patterns. In large cohorts (e.g., UK Biobank), the NOVA processing system was operationalised by mapping brief touchscreen items and limited 24 h recalls onto processing categories, an imperfect approximation of true processing exposure.	Misclassification of exposure of interest.	Repeated 24 h recalls; objective biomarkers; metabolomic dietary signatures.
Intermediate vs. hard endpoints	Reliance on LDL-C, BMI, fasting glucose as primary outcomes.	Surrogate change does not guarantee hard-outcome benefit.	Composite hard endpoints; pre-specify outcome hierarchy.
Trial duration	Most trials 8–24 weeks.	Cannot assess long-term nutrient status, bone, sarcopenia, cognition, reproductive outcomes.	12–24 months minimum; embedded biobanking; long-term follow-up.
Within-pattern heterogeneity	“Vegan” includes whole-food and ultra-processed; “omnivorous” includes Mediterranean and Western.	Aggregated effect estimates obscure within-group heterogeneity.	Stratify by NOVA processing and hPDI/uPDI on BOTH sides of the comparison.
COI and funding source	Industry sponsorship or author-level commercial interests from any food, supplement, or dietary-pattern sector.	Selective reporting; biased framing; asymmetric interpretation.	Transparent funding and conflict-of-interest disclosure; protocol registration where applicable; independent replication; symmetric interpretation of competing dietary patterns.

**Table 4:** Nutrients requiring systematic attention in vegan and vegetarian diets, with sources, supplementation/fortification guidance, and monitoring.

Nutrient or Compound	Nutritional Relevance in Vegan/Vegetarian Diets	Dietary Sources and Bioavailability Strategies	Supplementation and Fortification Guidance	Monitoring Parameters When Indicated
Vitamin B12 [12,45]	Not reliably present in unfortified plant foods.	Fortified plant beverages; fortified cereals; nutritional yeast (variable).	Yes—non-negotiable.	Serum B12, methylmalonic acid, homocysteine.
Iron [11,13,14]	Lower theoretical bioavailability of non-haem iron; phytate-rich matrix; recent evidence of physiological adaptation in long-term vegans.	Legumes, whole grains, seeds, tofu, with vitamin-C-rich foods; soaking/sprouting/fermentation.	Sometimes (menstruating women, athletes, pregnancy).	Ferritin, transferrin saturation, complete blood count.
Zinc [11,47]	Lower intake and bioavailability; phytate inhibition.	Legumes, seeds, whole grains, tempeh, nuts.	Sometimes (growth, pregnancy).	Dietary assessment; serum zinc.
Iodine [3,11]	Low intake without iodised salt, dairy, or seafood.	Iodised salt; controlled seaweed intake.	Often advisable, especially pregnancy.	Urinary iodine, TSH if symptomatic.
Calcium [36]	Lower intake; lower bioavailability from oxalate-rich greens.	Fortified plant beverages; low-oxalate greens; calcium-set tofu; almonds.	Sometimes.	Dietary assessment; DEXA across lifespan.
Vitamin D [3,11]	Few plant sources; endogenous synthesis variable.	Fortified foods; UV-exposed mushrooms.	Often required; vegan D3 (lichen) available.	25-hydroxyvitamin D.
EPA/DHA [11,48,49]	Limited and variable ALA conversion.	Microalgae-derived DHA supplements; ALA from flax, chia, walnuts.	Recommended, especially pregnancy/lactation.	Omega-3 index where available.
Protein quality [15,16,17,37,51]	Lower DIAAS; lower leucine/lysine per gram; but recent evidence shows no impairment of MPS or strength when planned.	Combine sources (legumes + cereals); soy, pea, mycoprotein; isolates when needed.	Often higher total protein recommended; isolates useful in older adults and athletes.	Total intake monitoring; lean mass.
Creatine, carnosine, taurine [20]	Absent or very low in vegan diets.	Supplementation only (no reliable plant source).	Creatine may be considered in athletes, older adults, or performance/cognitive contexts; others selectively.	Performance, cognition, muscle function.
Choline [50]	Lower intake than omnivorous.	Soybeans, broccoli, quinoa.	Sometimes, particularly pregnancy.	Dietary assessment.

**Table 5:** The comparative profile of five archetypal dietary patterns by quality, processing, animal-food inclusion, and nutrient planning requirements. Row shading denotes cardiometabolic risk stage: red rows indicate adverse/high-risk profiles (the ultra-processed Western omnivorous and the poorly planned vegan diet, which share a comparably adverse profile), and green rows indicate favourable profiles (well-planned vegan, Mediterranean omnivorous, and minimally processed omnivorous).

Dietary Pattern	Plant-Food Density	Processing Level	Animal-Food Inclusion	Likely Cardiometabolic Profile	Principal Nutrients Requiring Planning	Supplementation/Fortification Need
Ultra-processed Western omnivorous	Low	High (ultra-processed)	Processed meats; sweetened or highly processed dairy products	Adverse (high CVD, T2D, obesity)	Excess sodium, added sugars, saturated fat; low fibre and micronutrient density	Variable; supplementation need depends on baseline diet quality and nutrient status
Poorly planned vegan	Variable	Often high (plant analogues, refined grains, sweetened beverages)	None	Adverse to neutral	B12, iron, zinc, iodine, EPA/DHA, calcium, vitamin D, protein quality	High; risk increases when supplementation and fortified foods are absent or inconsistent
Well-planned vegan	High	Low to moderate	None	Favourable (cardiometabolic markers; advantage shown in some direct comparisons with high-quality omnivorous comparators). However, this favourable profile is conditional on strict, sustained adherence and ongoing mandatory supplementation (notably B12 and DHA); it is not intrinsic to the vegan label but depends on consistent planning and monitoring.	B12 (mandatory), DHA, iodine, sometimes iron and zinc	High and explicit
Mediterranean omnivorous	High	Low	Fish, olive oil, eggs, dairy, limited red/processed meat	Favourable (PREDIMED, CORDIOPREV hard endpoints)	Few; possible iron in menstruating women	Low
Minimally processed omnivorous	High	Low	Fish, eggs, lean meat, dairy if tolerated; limited red and processed meat	Plausibly favourable when plant-rich and minimally processed, but with less direct hard-outcome RCT evidence than the Mediterranean pattern	Few pattern-specific nutrients of concern; adequacy depends on quality of execution	Variable; usually lower reliance on mandatory supplementation than strict vegan diets

**Table 6:** Evidence map by outcome domain, principal studies, direction of effect, and simple certainty appraisal.

Outcome Domain	Evidence Type	Principal Studies (Refs.)	Direction	Certainty (Consistency/Design/Comparator/Duration/Endpoint)
Cardiovascular events	Cohort + RCT (Mediterranean only)	EPIC-Oxford [6]; PREDIMED [21]; CORDIOPREV [22]	Plant-rich diets favourable for IHD; vegetarians higher haemorrhagic stroke risk; Mediterranean reduces hard cardiovascular events	Moderate-to-high for Mediterranean hard endpoints; moderate for vegetarian observational signals
LDL cholesterol and lipids	RCT (multiple)	Landry 2023 [27]; OMNIVEG [25]; CARDIVEG [23]; Barnard 2022 [24]	Vegan/vegetarian patterns tend to reduce LDL-C beyond or at least similarly to Mediterranean comparators in short-term trials	Moderate-to-high for short-term LDL-C direction; moderate for magnitude; low-to-moderate for long-term clinical inference
Body weight and adiposity	RCT + cohort	Landry [27]; Barnard [24]; CARDIVEG [23]	Plant-rich patterns tend to reduce weight; vegan interventions often show greater short-term reductions	Moderate-to-high for short-term weight change; low-to-moderate for long-term weight maintenance
Type 2 diabetes and glycaemic control	Cohort + RCT	Landry [4,27]; Neufingerl [11]	Healthful plant-rich patterns are associated with lower T2D incidence and may improve short-term glycaemic markers	Moderate for observational associations; low-to-moderate for direct comparative trial evidence
Cancer incidence	Cohort + meta-analysis	Aune 2026 [34]; Dunneram 2026 [35]	Modest reduction overall; signals vary by cancer site and dietary subgroup	Low-to-moderate, limited by residual confounding, BMI, screening behaviour, exposure heterogeneity, and site-specific inconsistency
Bone/fractures	Cohort	EPIC-Oxford [36]	Higher fracture risk in vegans in EPIC-Oxford, partly attenuated by BMI, calcium intake, and protein intake	Low-to-moderate; mainly observational and potentially affected by residual confounding, BMI, supplementation, and baseline nutrient status
Muscle (MPS, strength)	RCT + meta-analysis	Domić 2025 [16]; López-Moreno 2025 [17]	No clear impairment in short-term studies when vegan diets are well planned and protein intake is adequate	Moderate for short-term outcomes; low-to-moderate for long-term sarcopenia, frailty, and functional endpoints
Exercise metabolism/performance	Controlled trial	OMNIVEG 2025 [26]	No apparent deterioration in fat oxidation, Fatmax, EE, HR, or RPE during short-term transition	Low-to-moderate; single short-term trial in healthy physically active men
Micronutrient adequacy/bioavailability	Systematic reviews + controlled trials	Neufingerl [11]; Pawlak [12]; Gibson [13]; López-Moreno [14]	B12, EPA/DHA, and iodine require planning; iron absorption shows adaptation in long-term vegans	High for B12/DHA; moderate for iron adaptation

## Data Availability

No new data were created or analysed in this study. Data sharing is not applicable.
